# Long non-coding HOXA-AS3 contributes to osteosarcoma progression through the miR-1286/TEAD1 axis

**DOI:** 10.1186/s13018-023-04214-5

**Published:** 2023-09-27

**Authors:** Xiangjun Xiao, Mingjiang Liu, Songlin Xie, Changxiong Liu, Xinfeng Huang, Xiongjie Huang

**Affiliations:** 1 Department of Hand and Foot Surgery, Nanhua Hospital Affiliated to Nanhua University, Hengyang, 421002 China; 2 Department of Orthopedic Trauma and Hand Surgery, Changsha Central Hospital Affiliated to Nanhua University, NO. 161 Shaoshan Nan Road, Changsha, 410018 China

**Keywords:** HOXA cluster antisense RNA 3, MiR-1286, Osteosarcoma, TEA domain family mem

## Abstract

Long non-coding RNA (lncRNA) HOXA cluster antisense RNA 3 (HOXA-AS3) regulates the progression of several types of human malignancy. However, the role and potential mechanism of HOXA-AS3 in osteosarcoma (OS) remain unknown. In this study, upregulation of HOXA-AS3 was observed in OS tissues and cell lines and associated with poor clinical outcomes. Silencing of HOXA-AS3 significantly inhibited the proliferation, migration and invasion of OS cells in vitro and suppressed the tumorigenesis of OS cells in vivo. Furthermore, knockdown of HOXA-AS3 inhibited the proliferation and migration of human umbilical vein endothelial cells (HUVECs) and epithelial-to-mesenchymal transition (EMT) in OS. Further investigation of this mechanism revealed that HOXA-AS3 could directly upregulate the expression of TEAD1 via its competing endogenous RNA (ceRNA) activity on miR-1286. This study clarified the oncogenic roles of the HOXA-AS3/miR-1286/TEAD1 axis in OS progression, suggesting a novel therapeutic target for OS.

**Supplementary Information:**

The online version contains supplementary material available at 10.1186/s13018-023-04214-5.

## Introduction

Osteosarcoma (OS) is one of the most common bone malignancies and most frequently occurs in adolescents and young adults [1, 2]. OS expresses a high level of aggression and metastasis. At present, surgery along with chemotherapy or radiotherapy is the basic treatment for OS. Although new chemotherapeutics and surgery have demonstrated improvements in recent years, the 5-year survival rates are as low as 19% in patients with OS having metastasis [3]. This is majorly because of the molecular mechanisms underlying the pathogenesis of OS, which are complicated and poorly understood. Therefore, it is necessary to identify novel and effective therapeutic targets.

Long non-coding RNAs (lncRNAs) are genome transcripts > 200 nucleotides; however, they lack protein-coding function [4]. Several studies have demonstrated that lncRNAs modulate different types of human cancers [5, 6]. In recent years, various types of lncRNAs have been revealed to be associated with OS tumourigenesis [7, 8]. For example, LINC00619 acts as a tumour suppressor in OS cells through HGF-dependent PI3K-Akt signalling [9]. LncRNA Ewing sarcoma-associated transcript 1 (EWSAT1) acts as an oncogene to promote lung metastasis in OS via the EWSAT1/miR-24-3p/ROCK1 axis [10]. LINC00607 promoted tumour proliferation in OS that worked as an miR-607 sponge to upregulate E2F6 expression [11]. LncRNA HOXA cluster antisense RNA3 (HOXA-AS3) is located on chromosome 7p15.2. Studies have revealed that HOXA-AS3 plays a tumourigenic role in non-small-cell lung carcinoma and glioblastoma [12, 13]. However, the role of HOXA-AS3 in OS has not been investigated.

In this study, we analysed the Gene Expression Omnibus (GEO) database and discovered that HOXA-AS3 was significantly upregulated in OS tissues. In addition, the expression of HOXA-AS3 was further confirmed in OS cell lines. Moreover, silencing of HOXA-AS3 inhibited cell proliferation, migration and invasion by sponging miR-1286 and acting as a competing endogenous RNA (ceRNA) for TEA domain family member 1 transcription factor (TEAD1). Therefore, this study indicated that HOXA-AS3 may be a promising therapeutic target for OS.

## Materials and methods

### GEO data analysis

The lncRNA expression data of 10 tumour tissues and 9 adjacent normal tissues were downloaded from the GEO (GSE126209) database. We used the edge R package with the threshold of |log (fold change) |> 1 and adjusted P-value < 0.05 to evaluate differentially expressed lncRNAs in normal as well as tumour tissues.

### Tissue samples

Forty OS tumour tissues and 20 non-tumour tissues were collected from patients with OS from 2017 to 2022 at the Changsha Central Hospital Affiliated to Nanhua University. All samples were obtained by biopsy before patients received radiotherapy or chemotherapy. This study was approved by the Ethical Committee of Changsha Central Hospital Affiliated to Nanhua University, and all patients gave informed consent.

### Cell culture

The human osteoblast cell line hFOB1.19 and OS cell lines MG63, HOS, 143B, Saos-2, SW1353 and U2OS were provided by Jennio Biotech (Guangzhou, China). All cells were cultured in Dulbecco’s Modified Eagle Medium (DMEM) (Invitrogen, Carlsbad, CA, USA) containing 10% foetal bovine serum (FBS) (Thermo Fisher Scientific, Waltham, MA, USA) and 100-U/mL penicillin/streptomycin. These cells were cultured under 5% CO_2_ in a 37 °C humidified atmosphere.

### Cell transfection

The short-hairpin RNA (shRNA) targeting HOXA-AS3 (sh-HOXA-AS3) and negative control (sh-NC) lentivirus were purchased from GenePharma (Shanghai, China). OS cells were treated with 8-mg/mL polybrene (Sigma) for 0.5 h before incubating them with sh-HOXA-AS3 and sh-NC lentivirus. After 12 h of incubation, 2-mg/mL puromycin (Sigma) was used to select stable cell lines. Cells were harvested for quantitative reverse transcription-polymerase chain reaction (qRT-PCR), which was performed to validate the transfection efficiency. The sequence of TEAD1 was sub-cloned into the pcDNA3.1 vector (Greenseed Biotech, Guangzhou, China) to construct overexpression vectors for TEAD1. The miR-1286 mimic, inhibitor and their negative controls were purchased from RiboBio (Guangzhou, China). The OS cell lines were transfected using Lipofectamine 2000 (Invitrogen).

### Quantitative reverse transcription-polymerase chain reaction

Cytoplasmic and nuclear RNA were isolated using a Cytoplasmic & Nuclear RNA Purification Kit (Norgen Biotek, Toronto, ON, Canada), whereas total RNA was isolated using the total RNA isolation TRIzol reagent (Invitrogen). Total RNA was reverse-transcribed to complementary DNA (cDNA) using the PrimeScript RT Reagent Kit (Takara, Dalian, China) and miRNA First-Strand Synthesis Kit (Takara). qRT-PCR was performed on the 7300 PCR System (Applied Biosystems). Data were analysed based on the 2^−ΔΔCt^ method. U6 and β-actin served as internal references. All primers used are as follows: miR-1286 RT primers: 5′-GTCGTATCCAGTGCAGGGTCCGAGGTATTCGCACTGGATACGACAGGGCT-3′, PCR primers: forward 5′-AAGCTGCAGGACCAAGATG-3′, reverse 5′-GTGCAGGGTCCGAGGT-3′; U6 primers: forward 5′-GCAAGGATGACACGCACAA-3′, reverse 5′-TGTGCGTGTCATCCTTGC-3′; HOXA-AS3 primers: forward 5′-GCTTCCACAATGTCCTGCTTC-3′, reverse 5′-TCAGGCTGCTGGGAAGAGTC-3′; TEAD1 primers: forward 5′-CGTTTCATCTGGTCAGTGGTTC-3′, reverse 5′-CCATCTCTCCAATCTACCCAAG-3′; β-actin primers: forward 5′-GAGGGAAATCGTGCGTGAC-3′, reverse 5′-TTCTGACCCATTCCCACC-3′.

### Western blot

Radioimmunoprecipitation (RIPA) lysis buffer was used for extraction of total protein from 143B and SW1353 cells (Beyotime Biotechnology, Shanghai, China), and protein concentration was quantified using a bicinchoninic acid (BCA) protein assay kit (Beyotime Biotechnology). The equivalent protein was subjected to electrophoresis on 10% sodium dodecyl sulphate–polyacrylamide gels (SDS-PAGE). Subsequently, the gels were transferred onto a polyvinylidene fluoride (PVDF) membrane. After blocking the membrane, they were incubated with primary antibodies such as TEAD1 antibody (1:2000, Abcam, Cambridge, MA, USA) and glyceraldehyde 3-phosphate dehydrogenase (GAPDH) antibody (1:5000; Abcam) at 4 ˚C overnight, followed by incubation with the secondary antibody (1:5000; Abcam) at room temperature for 1 h. The protein bands were visualised using enhanced chemiluminescence (ECL).

### Cell counting kit-8 assay

143B and SW1353 cells were seeded at a density of 1 × 10^4^ cells per well into 96-well plates after transfection. Cell viability was quantified according to the instructions of a cell counting kit-8 assay (CCK-8 assay) (Sigma-Aldrich) at 0 h, 24 h, 48 h and 72 h. The optical density (OD) was determined on a microplate reader (Thermo Fisher Scientific, Waltham, MA, USA) at 450 nm.

### 5-ethynyl-2′-deoxyuridine assay

The 5-ethynyl-2′-deoxyuridine (EdU) incorporation assay was performed using an EdU cell proliferation kit (Beyotime Biotechnology). Briefly, OS cells were seeded in 12-well plates 48 h later, and the cells were incubated with EdU solution for 2 h. Thereafter, cells were fixed with 4% paraformaldehyde. Thereafter, cells were stained with 4′,6-diamidino-2-phenylindole (DAPI) for 30 min. A microscope and the MetaXpress software (Molecular Devices, Sunnyvale, CA, USA) were used to acquire images.

### Transwell migration and invasion assays

For cell migration and invasion assays, cells were seeded in a 24-well transwell chamber (Corning Incorporated, Corning, NY, USA) with 8-μm pores coated with/without Matrigel (Corning Incorporated). The upper chamber was seeded with transfected cells in a serum-free medium, whereas a medium containing 10% FBS was added to the lower chamber. After 24 h, cells on the top layer of the membrane were removed. The migrated or invaded cells were fixed with 4% paraformaldehyde, stained with 1% crystal violet and observed under an inverted microscope (Olympus, Tokyo, Japan).

### Dual-luciferase reporter assay

The 3′-UTR fragment of TEAD1 and HOXA-AS3 containing wild-type (wt) or mutated (mut) miR-1286 binding sequences (TEAD1-3'UTR-wt, TEAD1-3'UTR-mut, HOXA-AS3-wt or HOXA-AS3-mut) was integrated into psiCHECK-2 luciferase reporter vectors (Promega, Madison, WI, USA). 293 T cells were maintained in 24-well plates and co-transfected with miR-1286 mimics and TEAD1-3'UTR-wt, TEAD1-3'UTR-mut, HOXA-AS3-wt or HOXA-AS3-mut luciferase reporter vector using Lipofectamine 2000 reagent (Invitrogen). After 48 h, the luciferase activity was determined using a dual-luciferase reporter assay system (Promega, USA) according to the manufacturer’s protocol. Relative Renilla luciferase activity was normalised to firefly luciferase activity.

### RNA immunoprecipitation assay

Cells were lysed using RIPA lysis buffer containing a protease inhibitor cocktail. The cell extraction was incubated with A/G magnetic beads that were conjugated with antibodies against Argonaute 2 (Ago2) or normal immunoglobulin G (IgG). The magnetic bead–protein complexes were incubated with Proteinase K to digest protein for the isolation of immunoprecipitated RNA, which was subjected to qRT-PCR to detect the expression of HOXA-AS3 or miR-1286.

### Immunohistochemistry (IHC) staining

Tissues were fixed with 4% neutralised formalin before being dehydrated and embedding in paraffin. 4-μm-thick slices were dewaxed and rehydrated; antigen retrieval was done in citrate buffer. Tissue sections were probed with Ki67 antibody (1:200, Abcam) and then incubated with secondary antibody (1:3000, Abcam). DAB Kit (Beyotime Biotechnology) was used to observe immunoreactive signals, and the nuclei were counterstained with haematoxylin (Sigma-Aldrich).

### Mouse tumour xenograft model

BALB/c male nude mice (4–6 weeks old) were kept in normal housing conditions with consistent humidity (45–50%), constant temperature of 20–25 °C, and a 12 h light/dark cycle. The 143B cells (5 × 10^7^ cells/mL) infected with sh-HOXA-AS3 or sh-NC lentivirus were subcutaneously inoculated into the axilla of nude mice. Every five days following the injection, the tumour size was estimated. Tumour volume was calculated following the formula: *V* = length × width^2^/2. Thirty days late, the nude mice were euthanised. The weight of xenografts was examined and tested again; tumours from mice were removed and collected for photography, qRT-PCR, and immunohistochemistry.

### Statistical analysis

Statistical analysis was performed using the Student’s t-test using the SPSS version 21.0 software (SPSS Inc., Chicago, IL, USA). Data were presented as mean ± standard deviation (SD). P < 0.05 indicated a statistically significant difference.

## Results

### High expression of HOXA cluster antisense RNA 3 was observed in osteosarcoma and positively associated with poor prognosis

**Fig. 1:**
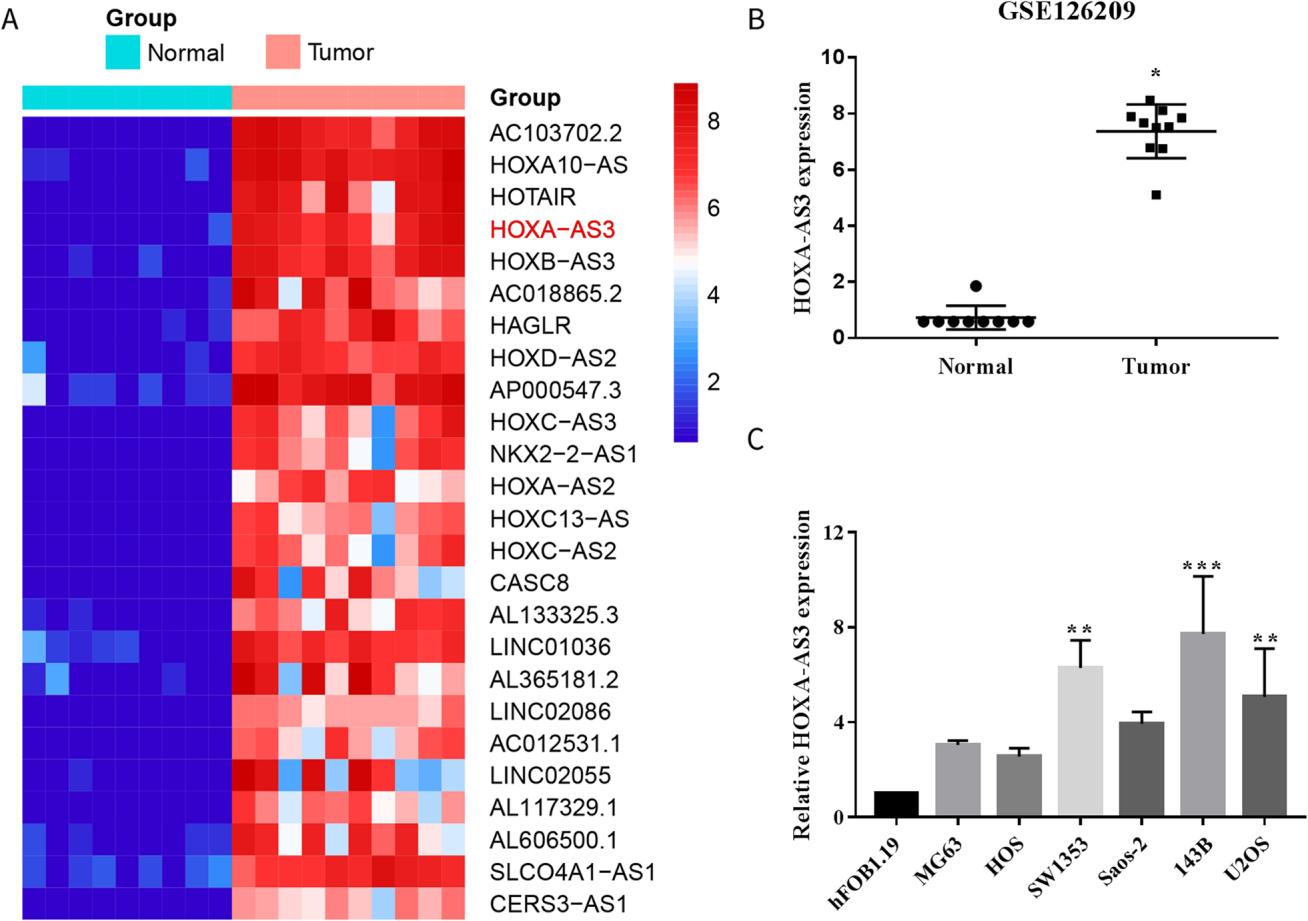
HOXA-AS3 was upregulated in OS. **A** Heatmap showing top 25 upregulated long non-coding RNAs in OS tissues in the GEO database. We ranked the fold change values for all lncRNAs in OS tissues. **B** Bioinformatic analysis revealed that HOXA-AS3 expression was high in OS. **C** HOXA-AS3 expression in OS cell lines was detected using qRT-PCR. Results are demonstrated as mean ± SD (*n* = 3; *P < 0.05, **P < 0.01, ***P < 0.001)

High-throughput sequencing data of 10 tumours and 9 adjacent normal tissues were obtained from the GEO database to screen for OS-associated lncRNAs (GSE126209). HOXA-AS3 was identified as an OS-associated lncRNA. Heatmap and volcano plot revealed that HOXA-AS3 expression was high in patients with OS (Fig. 1A, B, Additional file 1: Fig. S1A). By qRT-PCR analysis, elevated expression of HOXA-AS3 was observed in OS tissues compared to normal tissues (Additional file 1: Fig. S1B). Kaplan–Meier survival curves indicated that patients with higher expression levels of HOXA-AS3 had worse overall survival (Additional file 1: Fig. S1C). Based on clinical results, the expression of HOXA-AS3 in the OS cell lines 143B and SW1353 was compared with that of the normal osteoblast cell line hFOB1.19 (Fig. 1C). The results revealed that HOXA-AS3 expression was high in OS tissues and cell lines.

### Inhibition of HOXA cluster antisense RNA 3 suppressed the proliferation, migration and invasion of osteosarcoma cells

**Fig. 2:**
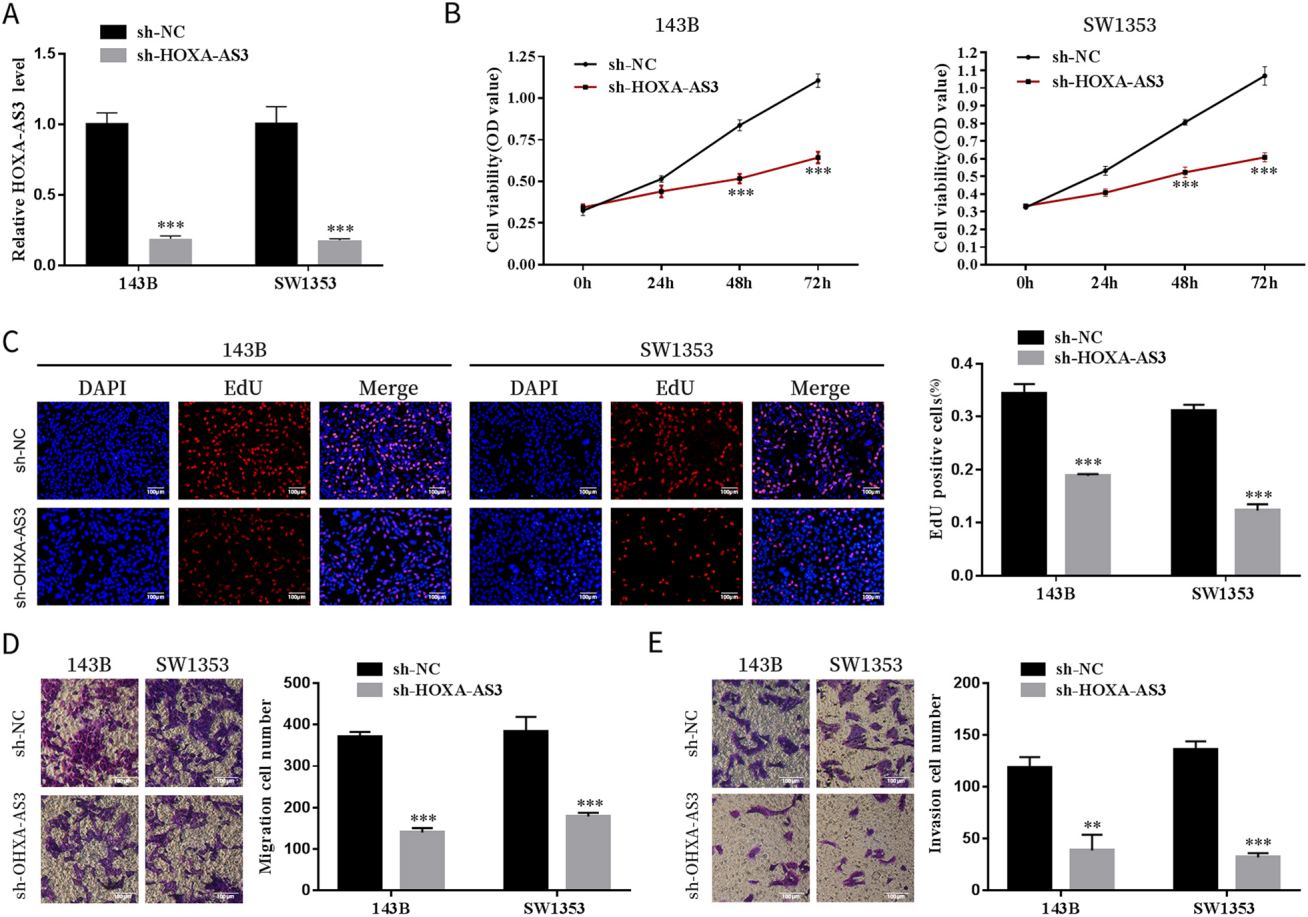
Inhibition of HOXA-AS3 suppressed the proliferation, migration and invasion of OS cells. **A** HOXA-AS3 was efficiently knocked down in 143B and SW1353 cells. **B** CCK-8 and EdU assays. **C** Revealed the effects of knockdown of HOXA-AS3 on 143B and SW1353 cell proliferation. **D** The transwell migration and invasion assays. **E** Revealed the effects of HOXA-AS3 silencing on the migration and invasion of OS cells. Results are expressed as mean ± SD (*n* = 3; _**_P < 0.01, ***P < 0.001)

Several in vitro experiments were performed to study the biological function of HOXA-AS3 in OS. 143B and SW1353 cells were transfected with sh-HOXA-AS3 or sh-NC lentivirus. The cells exhibited a relatively low level of HOXA-AS3 (Fig. 2A). It was discovered that the knockdown of HOXA-AS3 significantly inhibited OS cell viability (Fig. 2B). Consistent with the results of the CCK-8 assay, EdU assay further revealed that cell proliferation ability in the sh-HOXA-AS3 group was lower than that in the sh-NC group (Fig. 2C). Moreover, through transwell migration and invasion assays, we discovered that the migration and invasion capability of 143B and SW1353 cell lines was impaired after HOXA-AS3 silencing (Fig. 2D, E). These results indicated that HOXA-AS3 silencing inhibited OS progression in vitro.

### HOXA cluster antisense RNA 3 modulated endothelial cell function

**Fig. 3:**
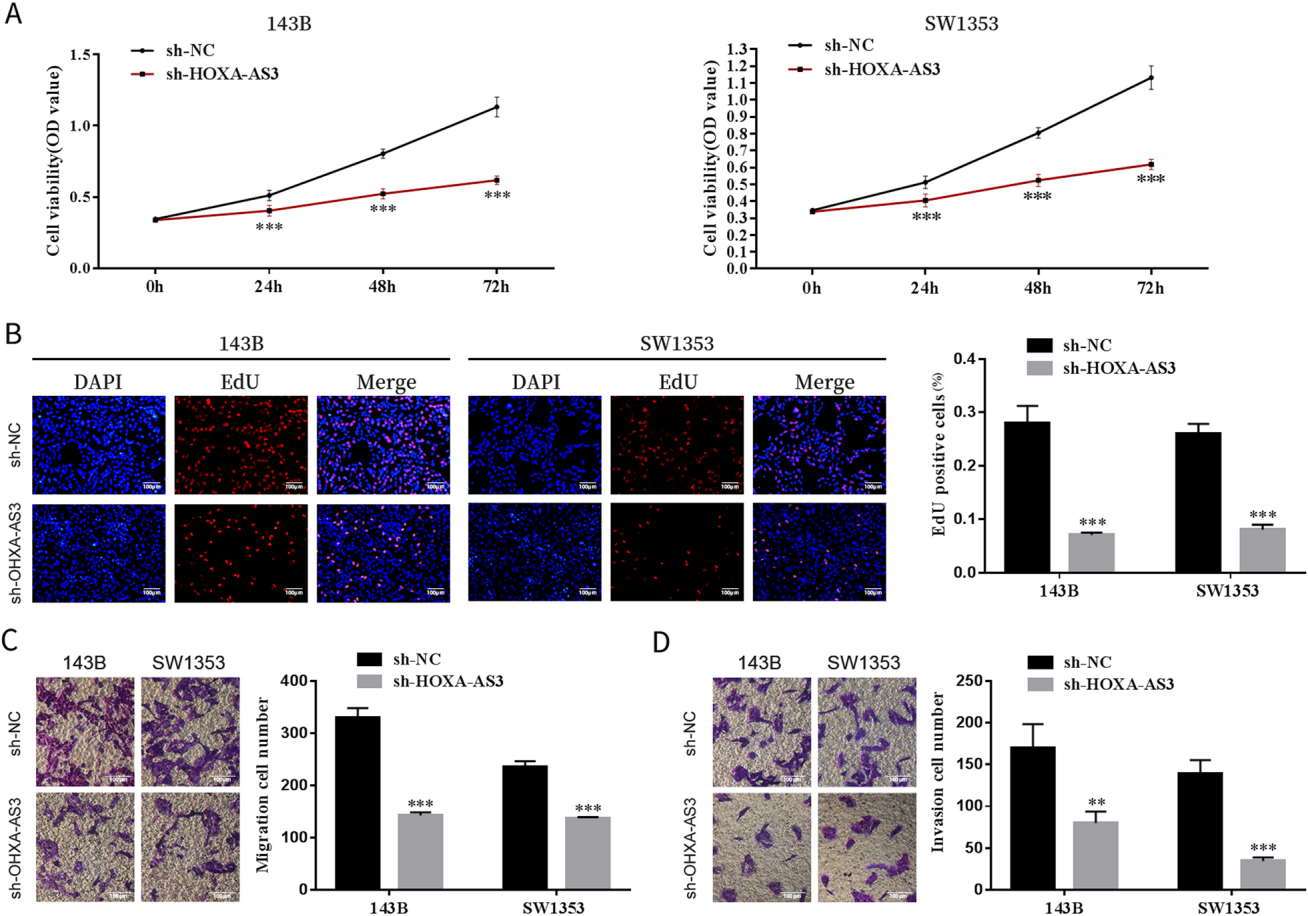
HOXA-AS3 modulated endothelial cell function. **A**, **B** Cell proliferation capability of HUVECs cultured with supernatants from sh-HOXA-AS3-transfected cells was assessed via the CCK-8 and EdU assays. **C** Relative cell migration and invasion **D** rates of HUVECs cultured with supernatants from sh-HOXA-AS3-transfected cells were measured. Results are expressed as mean ± SD (*n* = 3; **P < 0.01, ***P < 0.001)

Angiogenesis plays a vital role in tumourigenesis [14]. To examine the ability of endothelial cells in the anti-cancer effects caused by the inhibition of HOXA-AS3, we added cell culture supernatants from sh-HOXA-AS3-transfected cells to HUVECs. CCK-8 and EdU assays revealed that cell proliferation was impaired when HUVECs were cultured with supernatants from sh-HOXA-AS3-transfected cells (Fig. 3A, B). In addition, the migratory and invasion capabilities of HUVECs cultured with supernatants from sh-HOXA-AS3-transfected cells were decreased further (Fig. 3C, D). These data revealed that HUVECs treated with the supernatant of HOXA-AS3-knockdown OS cells had a decrease in proliferation, migration and invasion of endothelial cells.

### HOXA cluster antisense RNA 3 affected the epithelial-to-mesenchymal transition of osteosarcoma cells

**Fig. 4:**
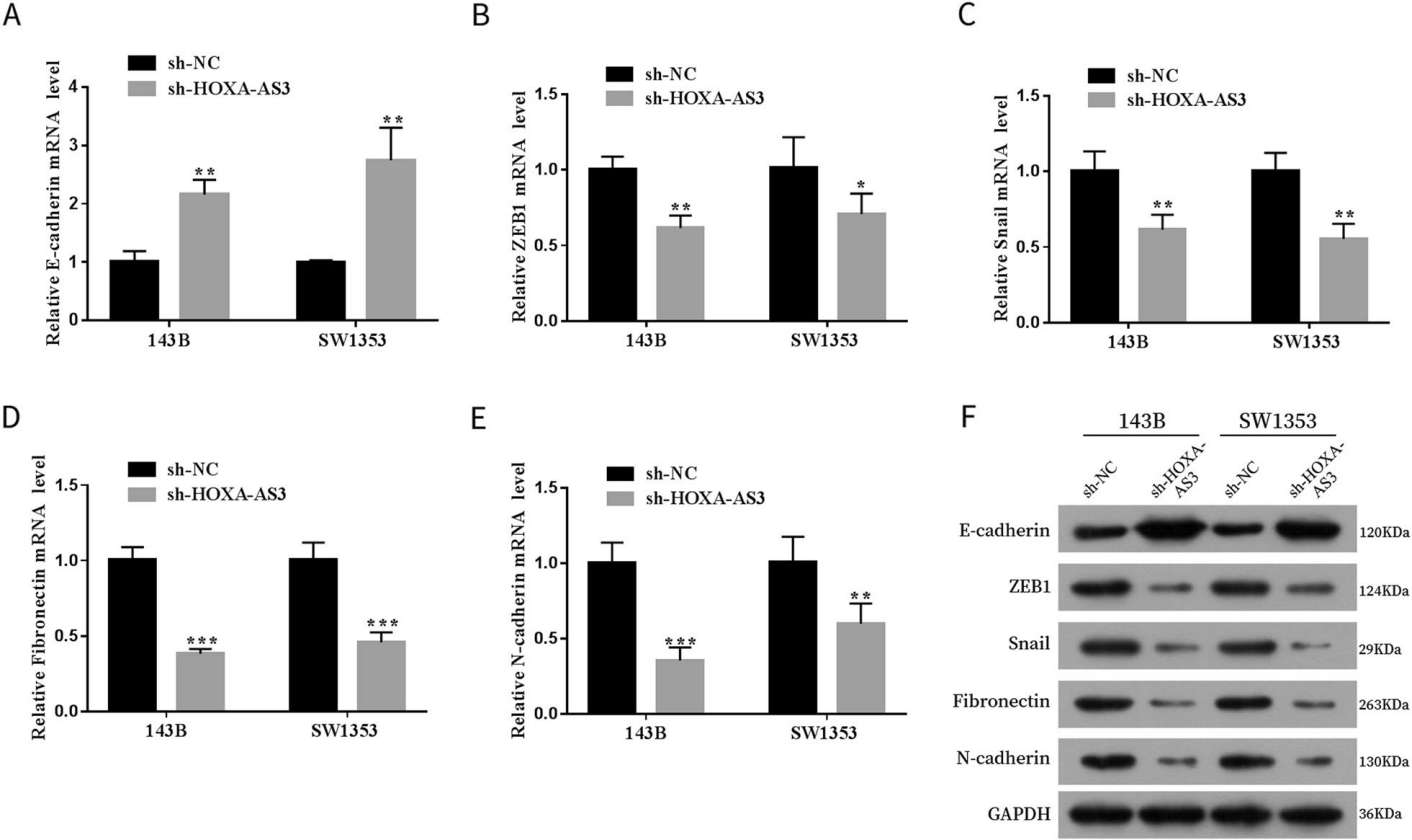
HOXA-AS3 affected the EMT of OS cells. **A–F** The mRNA and protein expression of EMT-related genes E-cadherin, ZEB1, SNAIL, fibronectin and N-cadherin in the sh-HOXA-AS3 and sh-NC groups was measured using qRT-PCR and western blot, respectively. Results are expressed as mean ± SD (*n* = 3; *P < 0.05, **P < 0.01, ***P < 0.001)

HOXA-AS3 affects the epithelial-to-mesenchymal transition (EMT) of OS, which plays a crucial role in tumour formation and progression [15, 16]. Several representative factors such as E-cadherin, ZEB1, SNAIL, fibronectin and N-cadherin [13, 17, 18] were used to investigate the influence of HOXA-AS3 on the EMT of OS. We discovered that ablating HOXA-AS3 increased the mRNA and protein expression of E-cadherin (Fig. 4A, F). Furthermore, other EMT-related genes including ZEB1, SNAIL, fibronectin and N-cadherin had the opposite reaction (Fig. 4B–F). These findings suggested that HOXA-AS3 knockdown hindered the EMT of OS cells.

### HOXA cluster antisense RNA 3 could bind to miR-1286 in osteosarcoma cells

**Fig. 5:**
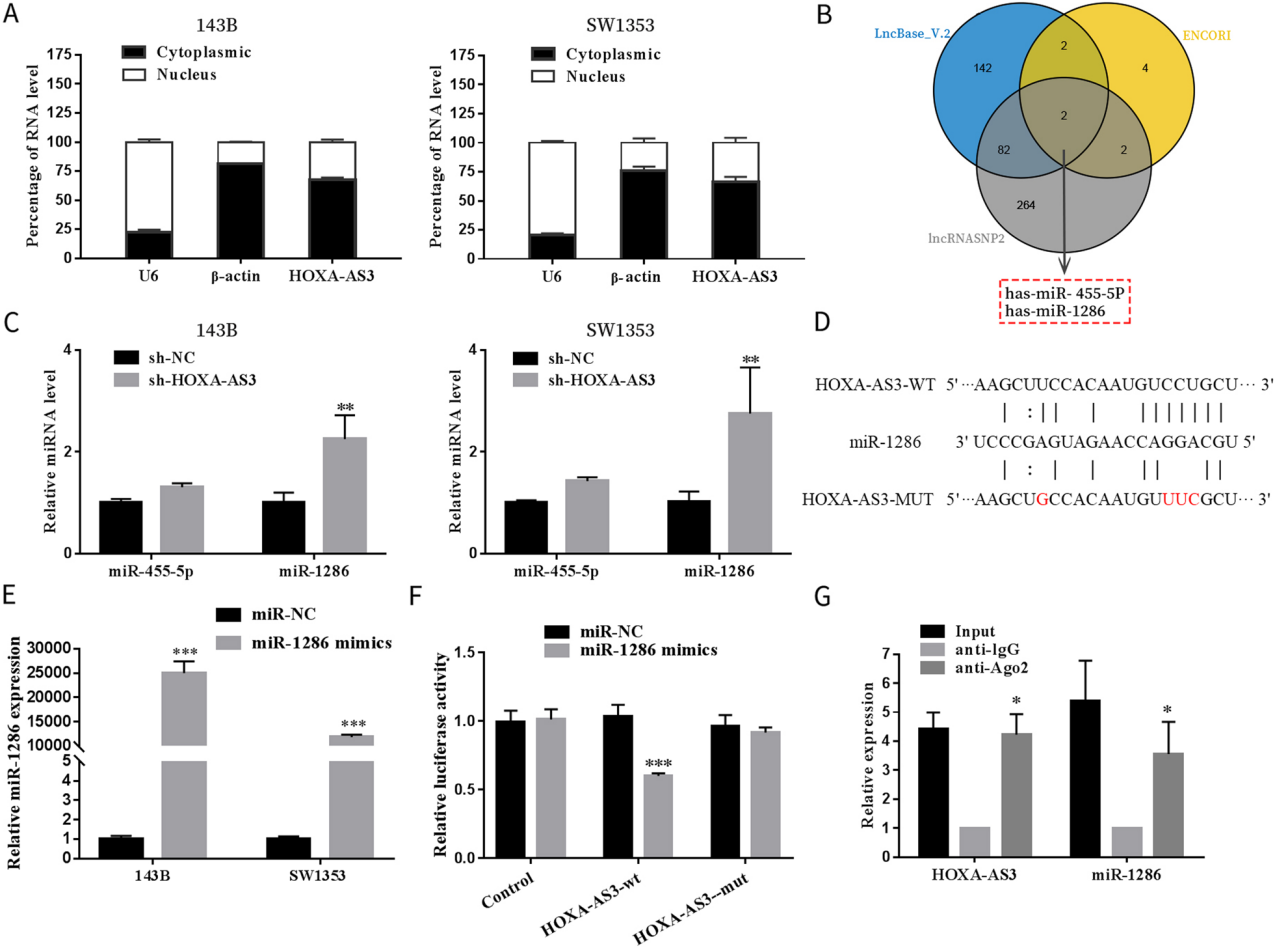
HOXA-AS3 binds to miR-1286 in OS cells. **A** Subcellular localisation of HOXA-AS3 in OS cells was assessed via qRT-PCR. **B** Binding of miRNAs to HOXA-AS3 was predicted using lncRNASNP2, lncBase V.2 and ENCORI databases. **C** qRT-PCR was performed to detect the expression of miRNAs in OS cells after HOXA-AS3 knockdown; **D** The binding site between HOXA-AS3 and miR-1286. **E** The relative expression of miR-1286 in OS cells transfected with control or miR-1286 mimics; **F, G** The relationship between HOXA-AS3 and miR-1286 was confirmed via dual-luciferase reporter and RIP assays. Results are expressed as mean ± SD (*n* = 3; *P < 0.05, **P < 0.01, ***P < 0.001)

To examine the mechanism of action of HOXA-AS3, we detected its subcellular localisation using qRT-PCR and discovered that HOXA-AS3 was primarily located in the cytoplasm (Fig. 5A). These results suggested that HOXA-AS3 may act as a miRNA sponge. Furthermore, mRNA prediction using lncBase V.2, ENCORI and lncRNASNP2 databases revealed miR-455-5p and miR-1286 as candidate targets of HOXA-AS3, and only miR-1286 was highly expressed in sh-HOXA-AS3-transfected cells (Fig. 5B, C). To determine whether miR-1286 is a target of HOXA-AS3, dual-luciferase reporter plasmids with HOXA-AS3-wt or HOXA-AS3-mut were established and transfected into 293 T cells. It was observed that miR-1286 markedly inhibited the luciferase reporter activity of HOXA-AS3-wt; however, it did not inhibit the activity of the HOXA-AS3-mut cells (Fig. 5D–F). Moreover, the RIP assay revealed that HOXA-AS3 and miR-1286 were enriched in the anti-Ago2 group (Fig. 5G). These data suggested that HOXA-AS3 could bind to miR-1286.

### miR-1286 inhibitor reversed the suppression of osteosarcoma cells induced by HOXA cluster antisense RNA 3 depletion

**Fig. 6:**
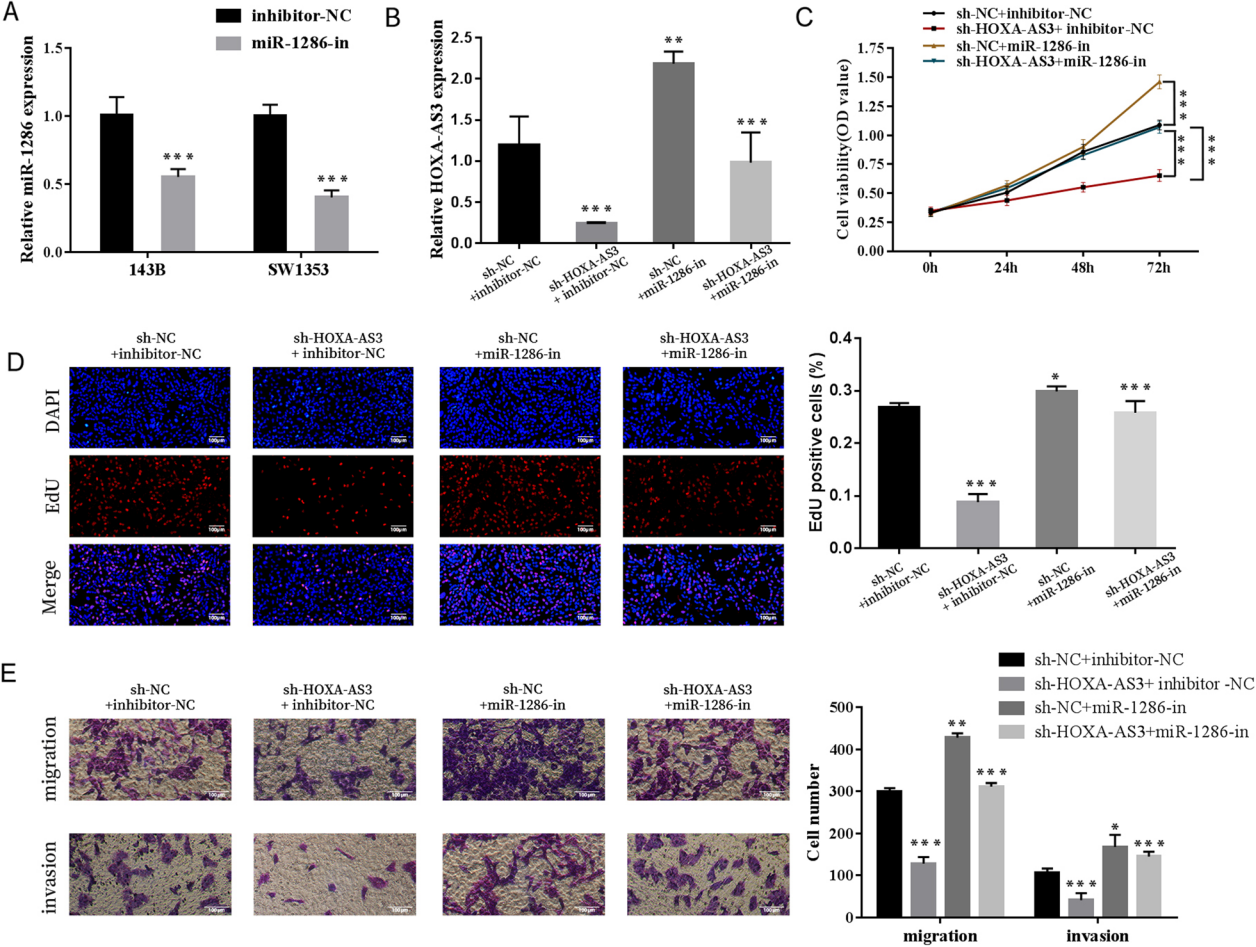
miR-1286 inhibitor reversed the suppression of OS cells induced by HOXA-AS3 depletion. **A** qRT-PCR analysis of miR-1286 expression in OS cells transfected with miR-1286 or control inhibitor. **B** The expression of miR-1286 was examined via qRT-PCR analysis in OS cells transfected with sh-HOXA-AS3 lentivirus, miR-1286 inhibitor (miR-1286-in) or both. **C** CCK-8 and EdU assays. **D** Were used to examine cell proliferation. **E** Transwell migration and invasion assays were performed to detect cell migration and invasion. Results are expressed as mean ± SD (*n* = 3; *P < 0.05, **P < 0.01, ***P < 0.001)

Given that miR-1286 functions as a target of HOXA-AS3, we further assessed whether HOXA-AS3 exerts biological functions via miR-1286. We performed a rescue experiment by transfecting sh-HOXA-AS3 lentivirus and miR-1286 inhibitor into 143B cells. As demonstrated in Fig. 6A, B the decreased expression of HOXA-AS3 was partially restored by miR-1286 inhibitor in sh-HOXA-AS3-transfected cells. In addition, miR-1286 silencing reduced the inhibitory effects of HOXA-AS3 knockdown on OS cell proliferation (Fig. 6C). Similarly, miR-1286 inhibitor partly restored the migration and invasion of OS cells reduced by silencing of HOXA-AS3 (Fig. 6D, E). These data suggested that HOXA-AS3 silencing regulated the bioactivity of OS cells through miR-1286.

### HOXA cluster antisense RNA 3 regulated the expression of TEA domain family member 1 through miR-1286

**Fig. 7:**
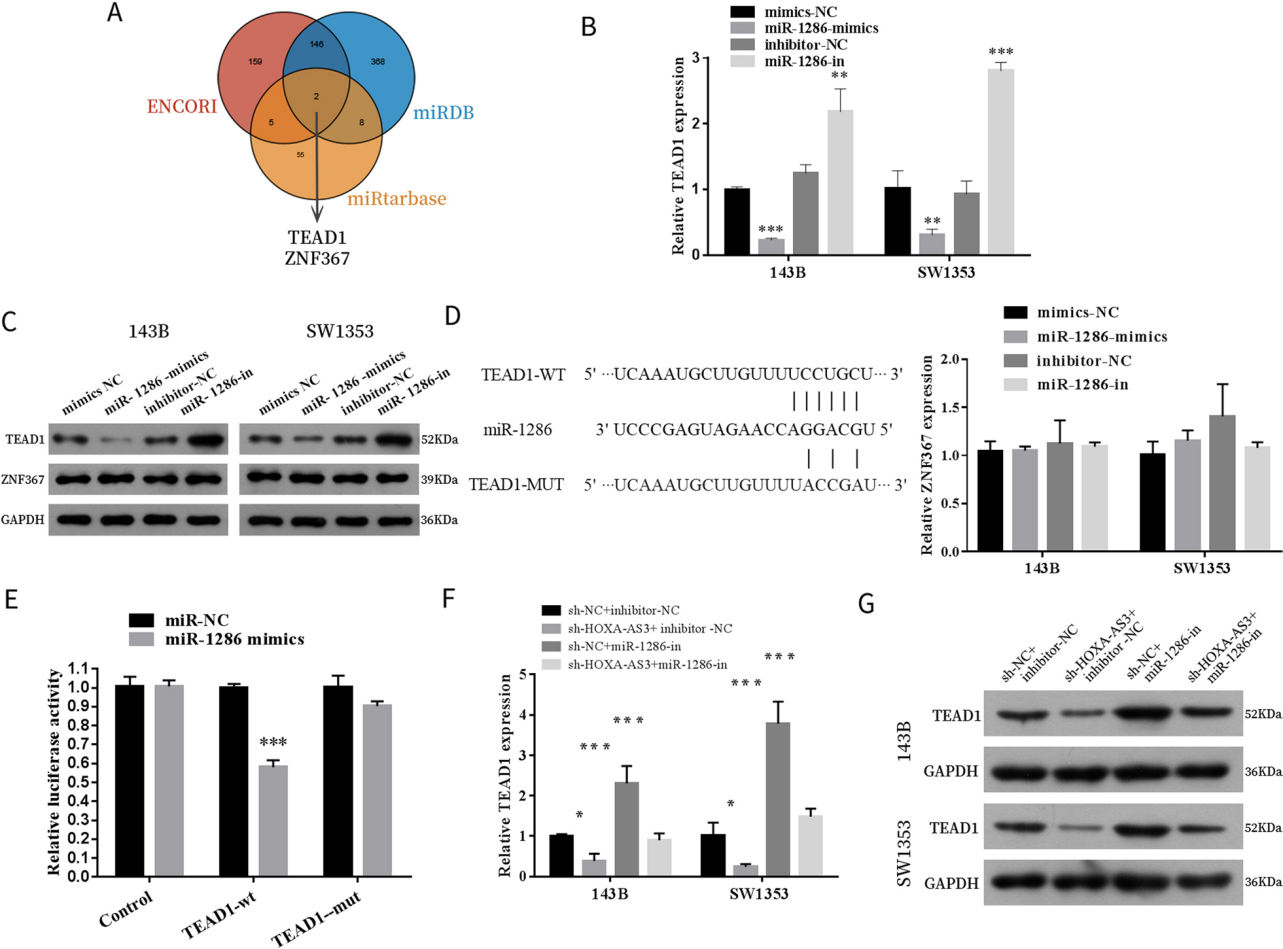
HOXA-AS3 regulates the expression of TEAD1 through miR-1286. **A** Predicted miR-1286 binding target genes via ENCORI, miRDB and miRtarbase. **B**, **C** The expression of TEAD1 and ZNF367 in 143B cells transfected with miR-1286 mimics or inhibitors was examined by qRT-PCR and western blot. **D** Predicted miR-1286 target sequences in 3'UTR of TEAD1 (TEAD1-3'UTR-wt) and mutant site in 3'UTR of TEAD1 (TEAD1-3'UTR-mut) are demonstrated. **E** Interaction between miR-1286 and TEAD1 was validated by a dual-luciferase reporter assay. **F**, **G** Relative mRNA and protein levels of TEAD1 in OS cells transfected with sh-HOXA-AS3 lentivirus, miR-1286 inhibitor or both. Results are demonstrated as means ± SD. *n* = 3; *P < 0.05, **P < 0.01, ***P < 0.001

ENCORI, miRDB and miRtarbase were used to predict the potential target genes of miR-1286; two genes (TEAD1 and ZNF367) were predicted as candidate targets of miR-1286 (Fig. 7A). According to qPT-PCR and western blot results, only TEAD1 was downregulated in miR-1286 mimic-transfected cells and upregulated in miR-1286 inhibitor-transfected cells (Fig. 7B, C). Furthermore, the dual-luciferase reporter assay demonstrated that miR-1286 overexpression significantly decreased the fluorescence intensity of TEAD1-3'UTR-wt vector instead of the TEAD1-3'UTR-mut vector (Fig. 7D, E). In the rescue experiment, miR-1286 inhibitor reversed the inhibition of TEAD1 expression after HOXA-AS3 knockdown in OS cells (Fig. 7F, G). These results suggested that HOXA-AS3 competitively binds to miR-1286 to promote TEAD1 expression.

### HOXA cluster antisense RNA 3 promoted the proliferation, migration and invasion of osteosarcoma cells via TEA domain family member 1

**Fig. 8:**
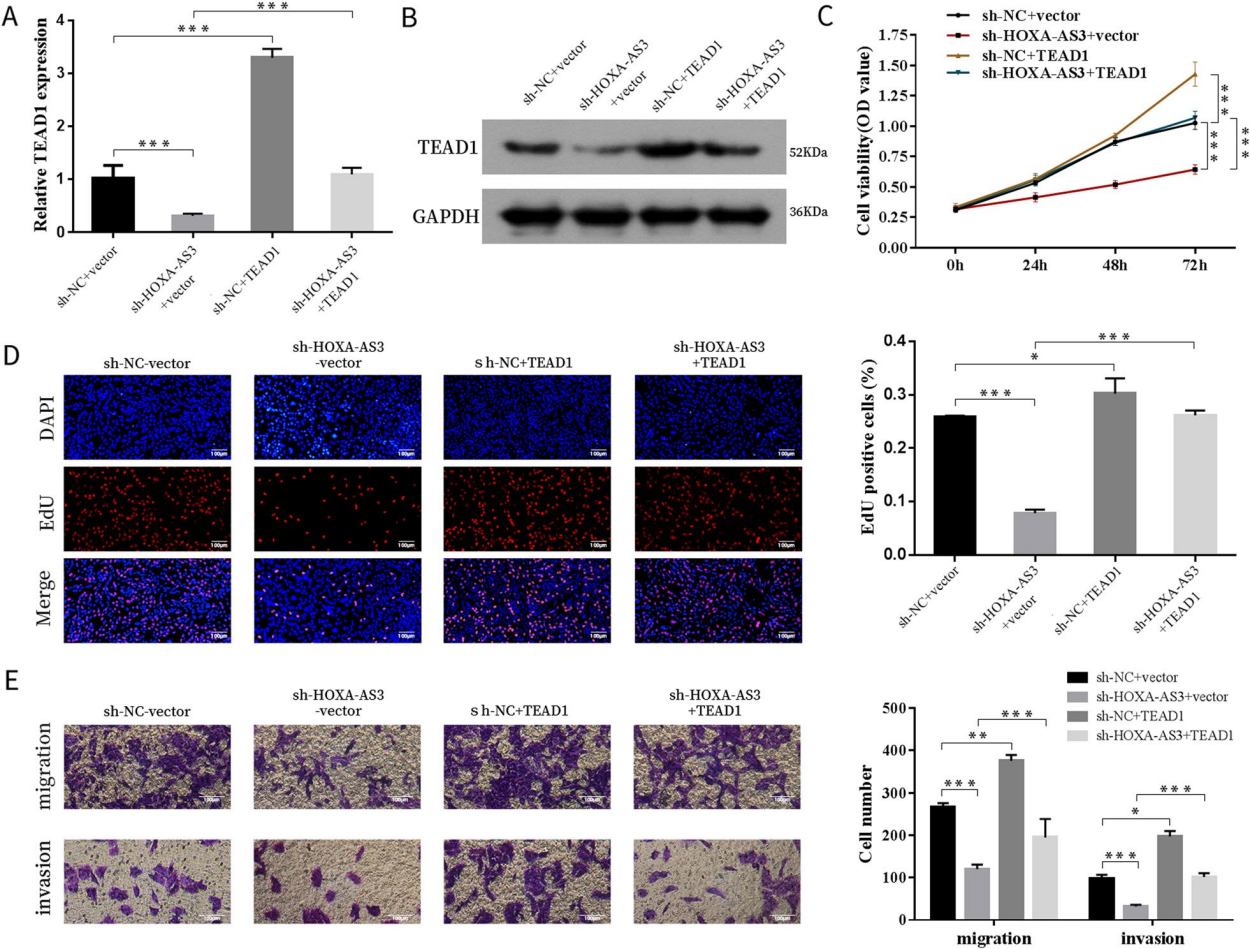
HOXA-AS3 promoted the proliferation, migration and invasion of OS cells via TEAD1. **A**, **B** The mRNA and protein expression levels of TEAD1 in 143B cells transfected with sh-HOXA-AS3 lentivirus, TEAD1 vector or both. **C-E.** Cell proliferation, migration and invasion abilities were measured via CCK-8, EdU, transwell migration and invasion assays, respectively. Results are expressed as mean ± SD (*n* = 3; *P < 0.05, **P < 0.01, ***P < 0.001)

To investigate whether HOXA-AS3 accelerates the progression of OS through TEAD1, rescue experiments were conducted. We co-transfected sh-HOXA-AS3 lentivirus and TEAD1 overexpression vector into 143B cells and discovered that TEAD1 overexpression attenuated the decrease in the expression of TEAD1 mRNA and protein caused by HOXA-AS3 knockdown (Fig. 8A, B). Furthermore, forced expression of TEAD1 partially rescued the effect of HOXA-AS3 depletion on the proliferation, migration and invasion abilities of OS cells (Fig. 8C–E). These results indicated that HOXA-AS3 exerted its function via TEAD1 in OS cells.

### HOXA-AS3 acted as an oncogene in xenograft tumour growth

**Fig. 9:**
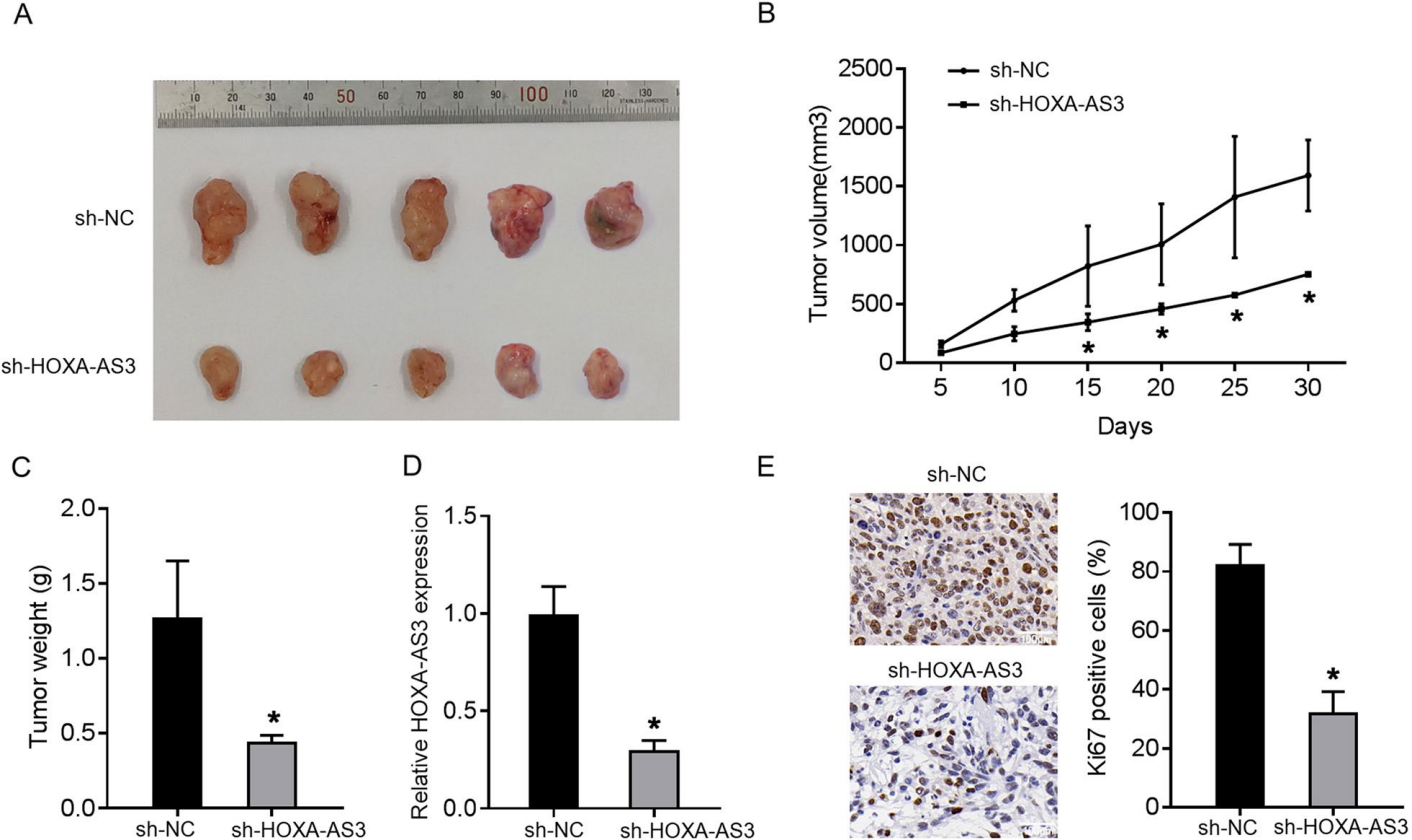
HOXA-AS3 acted as an oncogene in xenograft tumour growth. **A**–**C** Tumour development, volume, and weight in mice injected with sh-HOXA-AS3 or sh-NC lentivirus-infected 143B cells. **D** The relative expression of HOXA-AS3 in tumour tissues in sh-HOXA-AS3 injected group. **E** Immunohistochemistry staining was used to determine the expression of Ki67 in tumour tissues. Results are expressed as mean ± SD (*n* = 3; *P < 0.05, **P < 0.01, ***P < 0.001)

Then, the effects of HOXA-AS3 on OS tumour growth were further investigated. 143B cells infected with sh-HOXA-AS3 or sh-NC lentivirus were inoculated subcutaneously into mice. It was observed that tumour weight and volume were prominently decreased in the sh-HOXA-AS3 group compared to the control group (Fig. 9A–C). Furthermore, the expression of HOXA-AS3 in tumour tissues of the control group was significantly higher than that in the sh-HOXA-AS3 group (Fig. 9D). Furthermore, the expression of the proliferation marker Ki67 was reduced in the sh-HOXA-AS3 group (Fig. 9E). In addition, upregulation of TEAD1 in the sh-HOXA-AS3 group was observed (Additional file 1: Fig. S1D). These findings indicate that HOXA-AS3 accelerates tumour development in vivo.

## Discussion

lncRNAs have been reported to play an important role in biological processes [19], particularly in tumourigenesis [20]. HOXA-AS3 has been reported to regulate lineage commitment of mesenchymal stem cells [21]. Furthermore, in oncogenic contexts, HOXA-AS3 has been reported to be upregulated in gastric cancer tissues and cell lines and is correlated with tumour size, lymph node status and invasion depth [22]. Wang HC et al. reported that HOXA-AS3 promoted the proliferation, invasion and migration of glioblastoma cells in vitro and in vivo, which was discovered to be associated with poor survival prognosis in patients with glioma [13]. Moreover, HOXA-AS3 has been identified to modulate the resistance of non-small-cell lung carcinoma cells to cisplatin [12]. In this study, we discovered that HOXA-AS3 was upregulated in OS tissues and cell lines, which was consistent with the findings in lung adenocarcinoma tissues and A549 cells [23]. Moreover, high expression level of HOXA-AS3 is related to poor prognosis, and HOXA-AS3 knockdown inhibited the proliferation, migration and invasion of OS cells in vitro, as well as restrained tumour growth in vivo. These data suggested that HOXA-AS3 promoted GC progression.

As the first step in metastasis, tumour cells undergo a phenotypic transformation from epithelial to mesenchymal, which involves an important biological mechanism known as EMT [24, 25]. EMT has been reported to play a crucial role in enabling OS cells to migrate, disseminate, and establish metastatic growth at distant tissue sites [26, 27]. Consequently, inhibition of EMT holds great potential for suppressing tumour metastasis in OS [26, 27]. E-cadherin, N-cadherin, ZEB1, SNAIL and fibronectin are EMT-related genes [28, 29]. Therefore, we examined the expression of E-cadherin, N-cadherin, ZEB1, SNAIL and fibronectin to investigate the effects of HOXA-AS3 on the EMT of OS. Our results revealed that the downregulation of N-cadherin, ZEB1, SNAIL and fibronectin and the upregulation of E-cadherin was observed in sh-HOXA-AS3-transfected cells, suggesting that HOXA-AS3 silencing promoted the EMT process of OS. However, the mechanisms of HOXA-AS3 knockdown in the EMT of OS require further investigation.

Given the importance of angiogenesis in tumour progression [30], we studied the impact of HOXA-AS3 on HUVECs. The cell culture supernatants from sh-HOXA-AS3-transfected cells inhibited the proliferation, migration and invasion of HUVECs. However, factors that affect the HUVEC function require further investigation.

As ceRNAs, lncRNAs may serve as miRNA sponges by competitively binding with miRNAs to inhibit their targeting effects [31–33]. For instance, lncRNA BCRT1, which acts as a tumour promoter in breast cancer, can competitively bind to miR-1303 to prevent the degradation of its target gene PTBP3 [34]. In this study, we discovered that HOXA-AS3 was mainly localised to the cytoplasm where it can function as a ceRNA. These results revealed that HOXA-AS3 may affect OS by sponging a specific miRNA. Furthermore, we predicted the target miRNA of HOXA-AS3 using the lncBase V.2, ENCORI and lncRNASNP2 databases and confirmed miR-1286 as the target miRNA through dual-luciferase reporter and RIP assays. miR-1286 has been reported to play dual roles in cancers. A study discovered that miR-1286 was downregulated in OS tissues, and miR-1286 inhibitor promoted the proliferation, migration and invasion of OS cells, indicating that miR-1286 is a tumour suppressor of OS [35]. Similarly, miR-1286 has been reported to inhibit EMT and metastasis of cervical cancer cells [36]. However, Gao et al. revealed that miR-1286 promotes the development of lung cancer [30, 37]. In this study, we discovered that miR-1286 exhibited high expression in sh-HOXA-AS3-transfected cells. Moreover, the miR-1286 inhibitor partially reversed the inhibitory effects of HOXA-AS3 knockdown on OS cells, indicating that miR-1286 may be involved in the underlying mechanisms of HOXA-AS3.

As ceRNAs, lncRNAs can sponge miRNAs to modulate the expression levels of downstream target genes [17, 38, 39]. We used the ENCORI, miRDB and miRtarbase databases to predict that TEAD1 may work as a molecule for miR-1286. We validated this hypothesis by performing a dual-luciferase reporter assay. TEAD1 belongs to the TEAD family and participates in the Hippo signalling pathway, reportedly playing a crucial part in cell survival and proliferation [40]. Previous studies have discovered that TEAD1 is associated with cancers, including glioma [41], hepatocellular carcinoma [42] and clear cell renal cell carcinoma [43]. TEAD1 has been reported to be highly expressed in OS cells and tissues and accelerates multiple malignant phenotypes of OS cells including cell proliferation, apoptosis resistance and invasive potential [44–46]. Based on these studies, we further discovered that TEAD1 was underexpressed in sh-HOXA-AS3-transfected OS cells, and TEAD1 overexpression rescued the effects of HOXA-AS3 silencing on cell growth and metastasis. Altogether, our findings revealed that HOXA-AS3 functioned as a ceRNA to promote TEAD1-mediated proliferation and metastasis by binding to miR-1286 in OS.

**Fig. 10:**
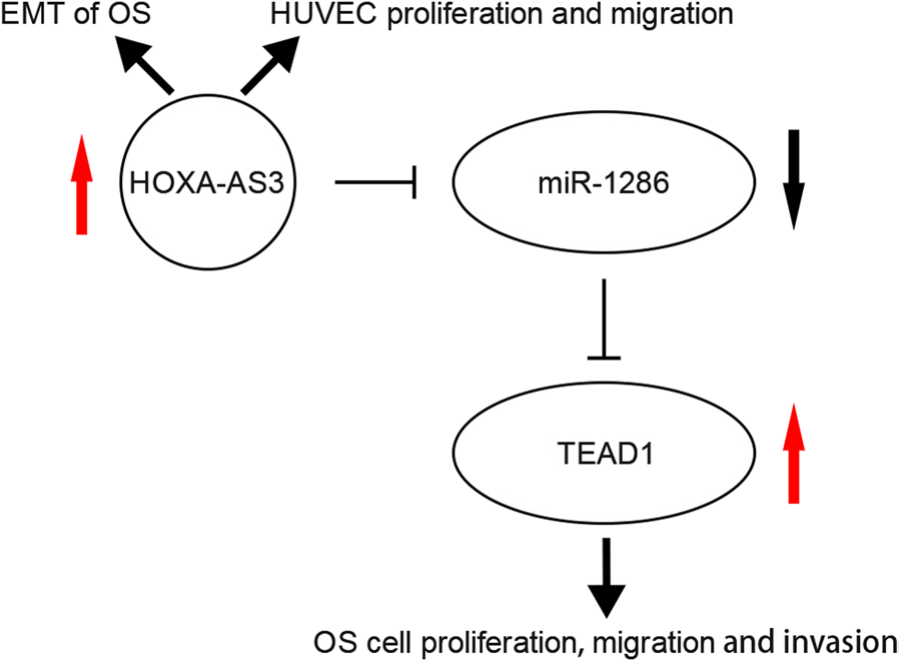
Schematic illustration of the role of HOXA-AS3 in OS cells

In conclusion, we discovered that HOXA-AS3 was upregulated in OS, and HOXA-AS3 promoted the proliferation, migration and invasion of OS cells. HOXA-AS3 served as an miR-1286 sponge to modulate the expression of TEAD1. Moreover, HOXA-AS3 knockdown suppressed EMT in OS and inhibited the proliferation and metastasis of HUVECs (Fig. 10). Therefore, HOXA-AS3 is a pivotal regulator of OS and may be a novel therapeutic target for OS.

### Supplementary Information

**Additional file 1: Fig. S1**. High expression of HOXA cluster antisense RNA 3 was positively associated with poor prognosis. **A** Volcano plot showing differentially expressed long noncoding RNAs in OS tissues. **B** The expression of HOXA-AS3 in OS tumour and paired normal tissues was detected by qRT-PCR. **C** Kaplan–Meier analysis of overall survival of OS patients. **D** TEAD1 protein expression in mice tissues was evaluated by Western blot. Results are expressed as mean ± SD (*n* = 4; **P < 0.01, ***P < 0.001).
