# Supplementary figures and images for: Long non-coding HOXA-AS3 contributes to osteosarcoma progression through the miR-1286/TEAD1 axis
Source: J Orthop Surg Res. 2023 Sep 27;18:730. doi: 10.1186/s13018-023-04214-5 (PMC10523635; doi:10.1186/s13018-023-04214-5)

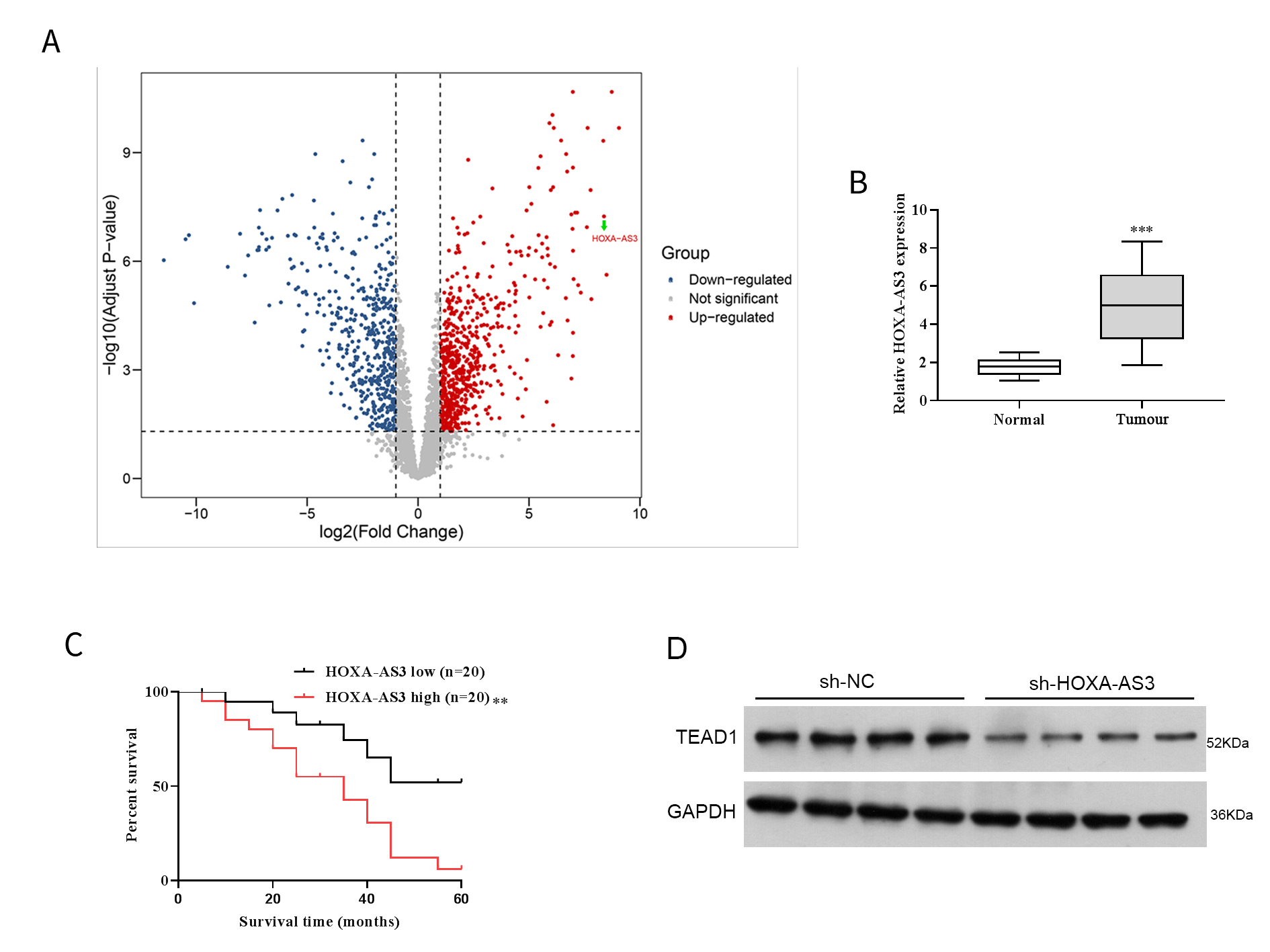

Supplement: Supplementary file 1 — Additional file 1: Fig. S1. High expression of HOXA cluster antisense RNA 3 was positively associated with poor prognosis. A Volcano plot showing differentially expressed long noncoding RNAs in OS tissues. B The expression of HOXA-AS3 in OS tumour and paired normal tissues was detected by qRT-PCR. C Kaplan–Meier analysis of overall survival of OS patients. D TEAD1 protein expression in mice tissues was evaluated by Western blot. Results are expressed as mean ± SD (n = 4; **P < 0.01, ***P < 0.001). [file 13018_2023_4214_MOESM1_ESM.jpg]
